# Prospective observational study of tofacitinib in ulcerative colitis – analysis of clinical data, fatigue and health-related quality of life during the induction phase

**DOI:** 10.1177/17562848251343427

**Published:** 2025-06-16

**Authors:** Lisa Nyberg, Jonas Halfvarson, Jonas Söderling, Ola Olén, Hans Strid, Charlotte R. H. Hedin, Sara B. Jónsdóttir, Henrik Hjortswang, Susanna Jäghult, Joseph C. Cappelleri, Dan Henrohn, Maria Seddighzadeh, Jan Marsal, Olof Grip, the SWIBREG ODEN Study Group

**Affiliations:** Department of Gastroenterology, Skåne University Hospital, Jan Waldenströms gata 16, 205 02 Malmö, Sweden; Department of Clinical Sciences, Lund University, Lund, Sweden; Department of Gastroenterology, Faculty of Medicine and Health, Örebro University, Örebro, Sweden; Division of Clinical Epidemiology, Department of Medicine Solna, Karolinska Institutet, Stockholm, Sweden; Division of Clinical Epidemiology, Department of Medicine Solna, Karolinska Institutet, Stockholm, Sweden; Karolinska University Hospital, Centre for Digestive Health, Stockholm, Sweden; Department of Gastroenterology, Dermatovenereology and Rheumatology, Centre for Digestive Health, Karolinska University Hospital, Stockholm, Sweden; Department of Medicine Solna, Karolinska Institutet, Stockholm, Sweden; Department of Gastroenterology, Dermatovenereology and Rheumatology, Centre for Digestive Health, Karolinska University Hospital, Stockholm, Sweden; Department of Health, Medicine and Caring Sciences, division of Gastroenterology and Hepatology, Linköping University, Linköping, Sweden; Department of Clinical Science and Education, Karolinska Institutet, Södersjukhuset, Stockholm, Sweden; Pfizer Inc., Statistical Research and Data Science Center, Groton, CT, USA; Department of Medical Affairs, Pfizer AB, Stockholm, Sweden; Department of Medical Sciences, Uppsala University, Uppsala, Sweden; Department of Medical Affairs, Pfizer AB, Stockholm, Sweden; Department of Clinical Sciences, Lund University, Lund, Sweden; Department of Gastroenterology, Skåne University Hospital, Malmö, Sweden

**Keywords:** fatigue, health-related quality of life, JAK inhibitor, tofacitinib, ulcerative colitis

## Abstract

**Background:**

Tofacitinib is a Janus kinase inhibitor for the treatment of ulcerative colitis (UC). Prospective real-world data are scarce.

**Objectives:**

To collect data on clinical outcomes, including health-related quality of life (HRQoL) and fatigue during treatment with tofacitinib.

**Design:**

This is a prospective observational multicentre study in Sweden. In this analysis, outcomes at weeks 2, 8 and 16 are reported.

**Methods:**

Patients with active UC confirmed with endoscopy or faecal calprotectin (FC) were enrolled during 2020–2023 when starting tofacitinib therapy.

**Results:**

In total, 103 patients were included. After 2 weeks of treatment, 50% (39/78) had achieved symptomatic response and at week 16, 39% (35/89) had achieved corticosteroid-free clinical remission according to the partial Mayo score. At week 16, a reduction in FC by ⩾50% was seen in 49% (35/71) and 24% (11/46) were in endoscopic remission. The frequency of arthralgia decreased from 29% (30/103) at baseline to 11% (10/89) at week 16. Regarding HRQoL at week 16; each of the four Short Health Scale dimensions (symptoms, social function, disease-related worry and general well-being) had improved by a median of 1 point (*p* < 0.01) and the European Quality of Life 5 Dimensions 5 Levels index improved from 0.80 to 0.87. Finally, the Inflammatory Bowel Disease Fatigue score measuring occurrence and severity showed an improvement with a decrease from 9 points at baseline to 6 at week 16 (*p* < 0.05).

**Conclusion:**

Induction therapy with tofacitinib therapy was associated with improvements in patient-reported outcome measures of symptoms, endoscopic activity, arthralgia, HRQoL and fatigue. These real-world data illustrate that tofacitinib is a fast-acting drug with broad therapeutic effects in UC.

**ClinicalTrial registration number:**

NCT04338204.

## Plain language summary

**Tofacitinib treatment in ulcerative colitis; results** **from treatment in the real world**

**What do we hope to find out?** If patients with Ulcerative Colitis and treatment with tofacitinib improve in various expressions of their disease. **Why was this research done?** Ulcerative colitis is an inflammatory condition of the bowel that often affects a person’s life substantially with an impact on quality of life. Symptoms of the bowel such as diarrhea, blood in stool and urgency are limiting symptoms but fatigue and joint pain may also be present. Some of the symptoms may be independent to intestinal inflammation. Before approval, drugs must demonstrate effectiveness in intestinal inflammation in randomized trials, but treatment and results in a clinical reality may differ from these settings. **What did we do?** Patients with active ulcerative colitis suitable for tofacitinib treatment were included in the study. Information concerning bowel symptoms, laboratory tests, quality of life and fatigue were collected at inclusion, week 2, week 8 and week 16. For some patients, endoscopic data was available. Information at follow up were compared to data before treatment for the same patient. Established questionnaires were used to quantify and evaluate the patient reported parameters. **What did we find out?** From 16 hospitals in Sweden, 103 patients were included. During the first 16 week of treatment, patients improved in symptoms. At week 16, 4/10 patients were in clinical remission. Quality of life improved in all measured aspects. Occurrence and severity of fatigue decreased by 1/3. Patients reporting joint pain at inclusion decreased to 1/3 at week 16. Some improvements were seen already week 2. These results indicate broad effects of tofacitinib.

## Graphical abstract

**Graphical abstract:**
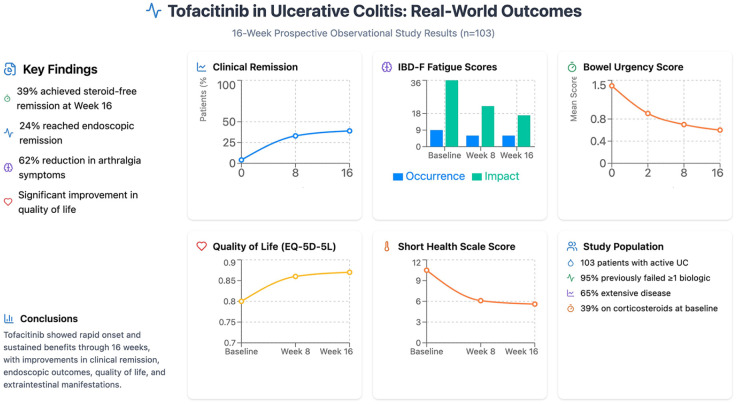

## Introduction

Ulcerative colitis (UC) is a chronic, relapsing inflammatory disease that affects the colon, resulting in both acute and chronic inflammation. The aim of treatment is to achieve long-term remission and to avoid complications, functional impairments and impact on daily life.^1^ Endoscopic healing and restoration of quality of life have been defined as important treatment targets in proposed management strategies.^2^

The use of Janus kinase inhibitors (JAKi) is now widespread and recommended in European and American guidelines as a second- or third-line therapy in patients with moderately to severely active UC.^3^ The ability of the JAKi tofacitinib to induce and maintain remission in UC has been well documented.^4^ However, by complementing pivotal registration studies with real-world studies that analyze a broader population, effects that have not been analyzed in randomized controlled trials (RCTs) can be captured. Real-world experience of tofacitinib is growing, but comprehensive prospective studies encompassing clinical outcomes, health-related quality of life (HRQoL) and extraintestinal manifestations (EIMs) are scarce, and detailed data on fatigue and bowel urgency are lacking.^5–7^

Tofacitinib is a synthetically produced small molecule that targets the intracellular JAK/signal transducer and activator of transcription (STAT) signalling pathway. Since a specific JAK protein is typically involved in the signalling of numerous cytokines, the inhibition of a single JAK protein may inhibit a whole group of cytokines, in contrast to biologics, which typically inhibit only one or two cytokines.^8^ The possibility to treat multiple expressions of a patient’s inflammatory condition with a single treatment is advantageous. Subgroup analyses from the tofacitinib registration studies suggested efficacy in inflammatory bowel disease (IBD)-associated arthritis.^9^ The pathogenesis of fatigue, a common symptom in IBD,^10,11^ is poorly understood but seems to be multifactorial, including increased levels of circulating cytokines as a likely important factor.^10,12^ However, when nutritional deficiencies and inflammatory activity are not present, it has been suggested that fatigue should be considered to be an independent EIM.^11^ Real-world data addressing the effects of tofacitinib in IBD-associated arthritis and other EIMs are limited.

The aims of this real-world study were to examine clinical outcomes after induction treatment with tofacitinib in UC and to investigate the effects on HRQoL, fatigue, bowel urgency and EIMs in parallel.

## Materials and methods

### Study design and data sources

This study (ODEN; Observational study of tofacitinib in UC in Sweden ClinicalTrial.gov No. NCT04338204) is a prospective multicentre study conducted at 16 Swedish sites, including both secondary and tertiary referral centres. Patients were included between August 2020 and September 2023. Patients with active UC starting tofacitinib treatment were eligible for inclusion. The reporting of this study conforms to the Strengthening the Reporting of Observational Studies in Epidemiology (STROBE) statement (Supplemental Material).^13^

**Figure 1.**
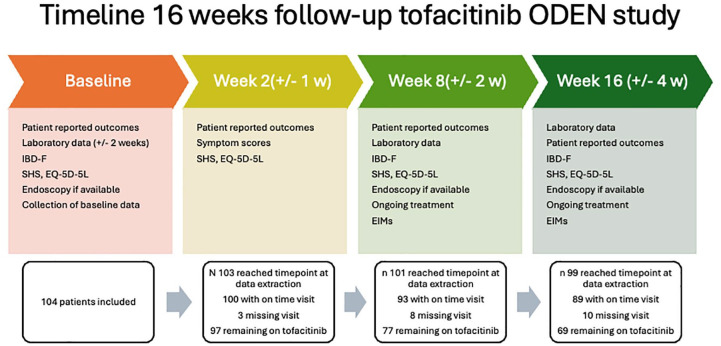
Timeline overview.

An electronic case report form (eCRF) connected to the Swedish Inflammatory Bowel Disease Registry (SWIBREG), with the possibility to retrieve data from the registry, as well as entering study-specific data, was used. Data were collected at baseline, week 2 (±1), 8 (±2) and 16 (±4) Figure 1. In this planned interim analysis, outcomes at weeks 2, 8 and 16 are reported. Local laboratories were used for all analyses and the choice of analysis methods was not regulated by the protocol. Drug safety was not an objective in this study, as patients included in this study will also be investigated in a separate post-approval safety study (A3921344).

### Patient population

Patients with confirmed UC diagnosis in whom tofacitinib was deemed appropriate therapy by the treating physician were candidates for the study. The ambition was to enrol 120 patients during the pre-set inclusion period. A power calculation estimated that a clinically significant improvement in the primary outcome, partial Mayo score (p-Mayo), at week 52 would be detected with this population size. The clinical decision to initiate tofacitinib therapy was clearly separated from the option to be included in the study. To be enrolled, patients had to be ⩾18 years of age and have active disease, defined by a Mayo endoscopic score (MES) of ⩾2 or faecal calprotectin (FC) >250 mg/kg, no more than 4 weeks before inclusion. No requirements regarding disease extent, duration or previous treatments were specified.

The initiation of tofacitinib was made in accordance with the summary of product characteristics (SmPC) with an induction dose of 10 mg twice daily for 8 weeks. Further dosing and additional treatment were recommended in accordance with the SmPC but decided by the treating physician due to the observational nature of the study. All patients who started on treatment were followed irrespective of treatment discontinuation.

### Outcomes

The primary outcome of the full study is remission at week 52 according to the p-Mayo score. In this interim analysis, we reported secondary and exploratory outcomes at week 2, week 8 and week 16, including retention rates of tofacitinib, clinical response and remission rates as defined by the p-Mayo score and symptom score, corticosteroid-free remission and bowel urgency (see below for definitions). Further outcomes were response and remission as defined by endoscopy or FC, the proportion of patients with C-reactive protein (CRP) ⩾5 mg/L, the colectomy rate and tofacitinib dosing. Outcomes regarding HRQoL included change from baseline in the Short Health Scale (SHS), European Quality of Life 5 Dimensions 5 Levels (EQ-5D-5L) and Inflammatory Bowel Disease Fatigue scale (IBD-F) scores described below. Exploratory outcomes were rates of patients with EIMs at baseline with a reduction of EIMs at week 16 and the proportion of patients in remission with the composite endpoint of endoscopy and FC.

The p-Mayo score ranges from 0 to 9 and includes stool frequency, rectal bleeding and physician global assessment.^14^ Clinical remission was defined as p-Mayo ⩽1 points with 0 points regarding rectal bleeding. Clinical response was defined as a decrease in p-Mayo of ⩾2 points and a reduction of at least 25% in p-Mayo from baseline, with an accompanying decrease of ⩾1 point in the rectal bleeding sub-score or an absolute bleeding sub-score of ⩽1 point. Symptomatic disease activity was defined by the sum of the two Mayo sub-scores for stool frequency and rectal bleeding, which is equivalent to PRO-2, with a score ranging from 0 to 6.^15^ Symptomatic remission was defined as a score of 0, and symptomatic response as a decrease of at least 50%.^2^ For bowel urgency, the Simple Clinical Colitis Activity Index bowel urgency sub-score was used, ranging from 0 to 3.^16^

With FC, the proportion that responded with a >50% reduction from baseline and the proportion that had a value of less than 250 mg/kg was stated. Endoscopic remission was defined as a MES of 0. Endoscopic response was defined as an improvement in MES of >1 point. The exploratory endpoint endoscopy/FC was defined as Mayo endoscopic sub-score 0 or when not available, FC <100 mg/kg.

SHS is a disease-specific questionnaire measuring HRQoL in IBD patients.^17^ SHS investigates four different aspects of HRQoL: symptom burden, social function, disease-related worry and general well-being, each graded on a 6-grade Likert scale. Each question has six levels of response scored 0–5, where 0 represents no impairment and 5 represents severe impairment, generating a total score of 0–20.

The EQ-5D-5L (EuroQoL, 2019) is a widely applied generic measure of health status validated in an IBD population.^18^ It consists of two parts of which the first part, the descriptive system, assesses health, with five items where each item represents one dimension of health: mobility, self-care, usual activities, pain/discomfort and anxiety/depression. Each dimension comprises five levels of response: no problem (1), slight problems (2), moderate problems (3), severe problems (4) and unable/extreme problems (5). The scoring ranges from 1 to 5. The Swedish preference utility value set was used to measure the index score for this population.^19^ The second part of the questionnaire consists of a visual analogue scale (VAS) by which the patient rates his/her perceived health from 0 (the worst imaginable health) to 100 (the best imaginable health). An increase of 0.05–0.08 in the EQ-5D-5L index and of 4.2–14.8 in the EQ-VAS measure has been suggested as relevant improvements for patients with IBD.^20,21^

The IBD-F scale was used since it has been developed to measure fatigue specifically for IBD patients.^22^ It consists of three parts, two score sections and a free-text section. The first section (IBD-F1) consists of five questions that assess the frequency and severity of fatigue. Each of the questions has five levels of response scored 0–4, where 0 represents no fatigue and 4 represents severe fatigue, generating a total score of 0–20. The patients are asked to grade their current fatigue and their fatigue during the past 2 weeks. The second section (IBD-F2) was used only in patients reporting some level of fatigue in the first section. IBD-F2 consists of 30 questions rating the experience and impact of fatigue in various situations and aspects of everyday life during the past 2 weeks. Each of the questions has five levels of response scored 0–4 where 0 represents ‘none of the time’ and 4 ‘all the time’, generating a total score of 0–120. Higher scores indicate a greater impact of fatigue. The free-text section in the IBD-F scale was not used in this study.

### Statistics

All patients included in the study were subjected to follow-up regardless of treatment discontinuation. For dichotomous clinical outcomes, the denominator consisted of all patients with available data at the timepoint or who had discontinued therapy (all patients having discontinued treatment were considered non-responders). In the analysis of biochemical markers, treatment discontinuation classified the patients as non-improved/high CRP or high FC. Patients with missing visits or data were considered missing at random and not included in the analysis unless information concerning treatment discontinuation/colectomy was obtained, and, if so, they were classified as non-responders.

For the measures SHS, EQ-5D-5L and fatigue, per protocol analysis was applied and only patients still on tofacitinib treatment were included in the analysis as in previous similar studies.^23,24^ Patients with missing visits or data were considered missing at random and not included in the analysis. Paired *t*-test was used for mean differences, and McNemar’s test was used for proportion differences.^25^ All analyses were two-sided, and *p* values <0.05 or 95% confidence interval not including 1 indicated statistical significance. Analyses were conducted in SAS version 9.4 (SAS Institute, USA, North Carolina).

## Results

### Baseline characteristics

In total, 103 patients with active UC were included. All patients over the age of 18 starting on tofacitinib at the included sites may have been considered for inclusion, but eligibility was judged by the physician and the total number of patients considered is not known. Three patients withdrew consent during the 16 weeks presented here, and only data collected prior to withdrawal are included. Basic demographic and clinical characteristics are shown in Table 1. A majority of patients, 65% (67/103), had extensive disease. The proportion of patients who previously had failed at least one biologic was 95% (98/103), at least two biologics, 62% and 37% had failed three biologics or more. Two patients were naïve to both immunomodulators and biologics prior to initiating tofacitinib treatment. The proportion of patients receiving corticosteroid treatment at inclusion was 39% (40/103) with a median dose of 40 mg prednisolone or equivalent. Three patients (3%) had a history of colectomy and ileorectal anastomosis. All patients were started on a tofacitinib dose of 10 mg twice daily.

**Table 1.** Characteristics of the study population at the start of tofacitinib treatment.

Baseline characteristics	Outcome
Number of patients	103
Female gender %/(*n*)	41% (42)
Age, years, median (IQR)	38 (26–46)
Current smoker (%)/CR%	2/97
Body mass index, median (IQR)/CR%	24.2 (21.6–27.2)/97
UC disease	
Disease duration years, median (IQR)	6.0 (2.6–14.3)
Montreal classification %/(*n*)	
E1 Proctitis	6% (6)
E2 Left-sided colitis	29% (30)
E3 Extensive colitis	65% (67)
Disease activity	
Full Mayo score, mean (SD)/CR%	7.1 (2.3)/72
Partial Mayo score, mean (SD)/CR%	4.7 (2.0)/99
Symptomatic disease activity,^a^ mean (SD)/CR%	2.6 (1.6)/99
P-CRP mg/L, median (IQR)/CR%	4.0 (1.5–11.0)/99
F-Calprotectin mg/kg, median (IQR)/CR%	980 (522–2250)/90
Extraintestinal manifestations %/(*n*)	
Arthralgia	29% (30)
Erythema nodosum	0
Pyoderma gangrenosum	0
Uveitis/iritis	2% (2)
Primary sclerosing cholangitis	0
Comorbidity %/(*n*)	
Cardiovascular disease	13% (13)
Lung disease	5% (5)
Liver disease	3% (3)
Diabetes	9% (9)
Chronic inflammatory disease other than IBD	7% (7)
Concomitant medication %/(*n*)	
Corticosteroids	39%/(40)
5-Aminosalisylalic acid	39%/(40)
Previous medication % (*n*)	
Thiopurines	57% (59)
Any biologic drug	95% (98)
Adalimumab	66% (68)
Infliximab	56% (58)
Golimumab	4% (4)
Ustekinumab	18% (19)
Vedolizumab	42% (43)

CR reflects percentage of the whole group of 103 patients with data available for the individual parameter. Full coverage rate when not specified. *n* = sample size.

a The sum of the Mayo sub-score stool frequency and rectal bleeding.

CR, coverage rate; CRP, C-reactive protein; IQR, interquartile range; SD, standard deviation; UC, ulcerative colitis.

### Tofacitinib retention rates

The retention rates at week 8 and 16 were 83% (77/93) and 78% (69/89), respectively. Two patients underwent colectomy before week 8, and at week 16 the cumulative colectomy rate was 3% (three patients), whereas 8/89 patients (9%) had switched to ustekinumab, vedolizumab or a tumour necrosis factor (TNF) inhibitor. No patient was on combined therapy tofacitinib and biologic at week 16. Two patients stopped tofacitinib treatment due to intolerance. Among patients remaining on tofacitinib treatment after 16 weeks, 62% (43/69) were on tofacitinib 10 mg twice daily and 38% (26/69) had lowered the dose to 5 mg twice daily.

### Clinical disease outcomes

**Figure 2.**
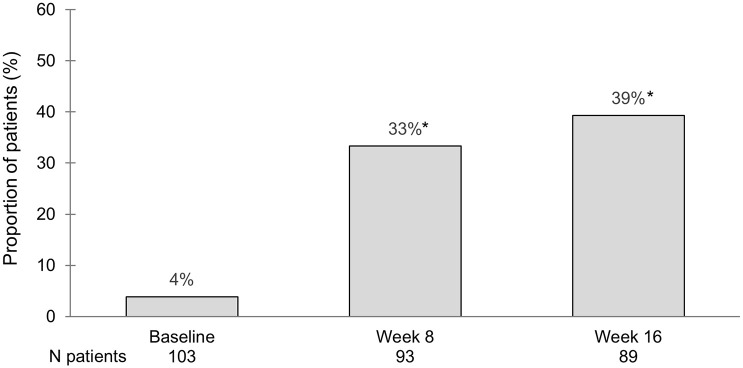
Patients in steroid-free remission defined by the partial Mayo score at follow-up in all patients. Patients with tofacitinib discontinuation are considered treatment failures and cannot achieve remission. *Significant changes from baseline using McNemar’s test for proportion differences (*p* < 0.05).

Outcomes at week 2, week 8 and week 16 are shown in Table 2 with accompanying baseline values. Mean p-Mayo scores at baseline, week 8 and week 16 were 4.7, 2.3 and 2.0, respectively, in the overall population regardless of ongoing treatment. Corticosteroid-free remission rates as measured by the p-Mayo score are demonstrated in Figure 2. The mean symptomatic disease activity, as measured by the sum of the Mayo sub-scores stool frequency and rectal bleeding, were at baseline and after 2 weeks 2.6 and 1.6, respectively. The rate of corticosteroid-treated patients dropped from 39% (40/103) at baseline to 14% (13/93) at week 8 and 8% (7/89) at week 16.

**Table 2.** Clinical outcomes at baseline and follow-up in all patients.

Clinical outcomes	Baseline	Week 2	Week 8	Week 16
Observed patients, *n*	103	103	101	99
Patients in remission defined by				
p-Mayo score, Prop % (CR%)	6 (98)	N/R	41* (87)	45* (87)
Symptoms (SFS/RBS), Prop % (CR%)	13 (99)	28* (87)	38* (89)	41* (87)
Patients with responses defined by				
p-Mayo score, Prop % (CR%)	N/A	N/R	83 (63)	77 (63)
Symptoms (SFS/RBS), Prop % (CR%)	N/A	50 (76)	59 (79)	57 (77)
Patients with no bowel urgency, Prop % (CR%)	15 (98)	34* (87)	39* (89)	42* (87)
Patients with Mayo endoscopic score ⩽1, Prop % (CR%)	8 (74)	N/R	33* (39)	44* (47)
Patients with F-calprotectin, Prop % (CR%)				
<250 mg/kg	12 (90)	N/R	51* (72)	45* (75)
Reduction ⩾50%	N/A	N/R	54 (68)	49 (72)
Patients with C-reactive protein ⩾5 mg/L, Prop % (CR%)	38 (99)	N/R	13* (83)	15* (81)

CR reflects the percentage of the whole group with data available for the individual parameter. All patients regardless of ongoing tofacitinib treatment are included. Patients with tofacitinib discontinuation are considered treatment failure and cannot achieve treatment success (response, remission, low calprotectin, low CRP or low endoscopic score in this analysis). *n* = sample size.

* Significant change from baseline using McNemar’s test for proportion differences, *p* < 0.05.

CR, coverage rate; N/A, not applicable; N/R, not reported; p-Mayo, partial Mayo; Prop, proportion of patients; RBS, rectal bleeding score; SFS, stool frequency score.

**Figure 3.**
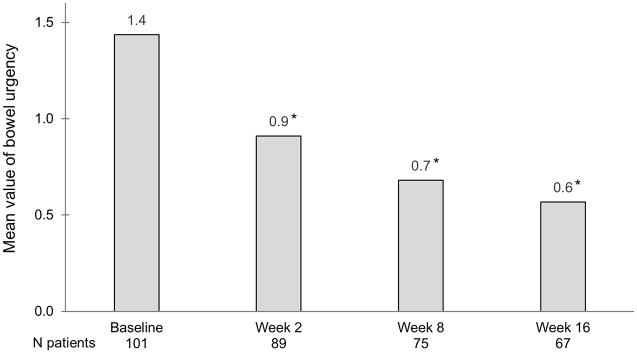
Bowel urgency at baseline and follow-up measured by the SCCAI urgency sub-score for patients with ongoing tofacitinib treatment. *Significant changes from baseline using paired *t*-test for mean differences (*p* < 0.05). SCCAI, simple clinical colitis activity index.

The proportion of patients that reported no urgency at follow-up increased significantly already at week 2 and continued to increase by nearly 3-fold compared to baseline at week 16 (Table 2). The mean urgency values for patients on tofacitinib at baseline and at 2, 8 and 16 weeks are shown in Figure 3.

The EIM arthralgia was present in 29% (30/103) of patients at baseline. At follow-up, 13% (12/93) and 11% (10/89) had remaining arthralgia at weeks 8 and 16, respectively. Of the patients with data on FC levels at baseline and at follow-up, the median (interquartile range) FC level (mg/kg) dropped from 980 (522–2250) at baseline to 188 (53–830) and 140 (29–640) at weeks 8 and 16, respectively. No patients had pyoderma gangrenosum, erythema nodosum or primary sclerosing cholangitis at any timepoint. Two patients had uveitis/iritis at baseline but none at week 8 or 16.

### Endoscopic outcomes

**Figure 4.**
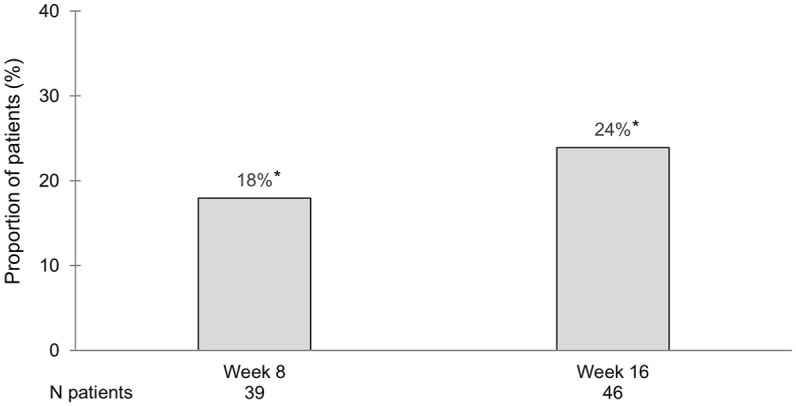
Patients in endoscopic remission defined by the Mayo endoscopic score of 0 in all patients. Patients with tofacitinib discontinuation are considered treatment failure and cannot achieve remission. *Significant changes from baseline using McNemar’s test for proportion differences (*p* < 0.05).

Endoscopic evaluation data was available for 74% (76/103) of patients at inclusion and 39% (39/101) and 46% (46/99) at week 8 and week 16, respectively. The proportions of patients achieving endoscopic remission at 8 and 16 weeks of treatment are shown in Figure 4. Endoscopic response rates were somewhat higher, 46% (17/37) and 45% (19/42) of patients at 8 and 16 weeks, respectively. The endpoint of endoscopy when available (MES 0) or FC <100 mg/kg showed remission rates of 30% (24/81) at week 8 and 38% (31/82) at week 16 with a data coverage rate of 80% (81/101) at week 8 and 83% (82/99) at week 16.

### HRQoL and fatigue outcomes

Mean outcomes regarding HRQoL are shown in Table 3. Investigating the effects of tofacitinib treatment on HRQoL, we analyzed the four dimensions of the HRQoL index SHS (symptoms, social function, disease-related worry and general well-being) and the SHS total score. Significant decreases in the scores of all of the four dimensions and the SHS total score at week 8 compared to the baseline values (Table 3). These improvements were maintained through week 16 with a tendency toward further amelioration (Table 3).

**Table 3.** Health-related quality of life outcomes at baseline and follow-up in patients with ongoing tofacitinib treatment.

Outcomes	Baseline *n* = 103	Week 8 *n* = 77	Week 16 *n* = 69
	Mean values (SD)	Mean change from baseline (95% CI)	Mean change from baseline (95% CI)
SHS^a^			
SHS total score	10.5 (4.2)	−4.4 (−5.2; −3.5)*	−4.9 (−5.9; −3.8)*
SHS sub-scores			
Symptoms	2.7 (1.3)	−1.3 (−1.5; −1.0)*	−1.3 (−1.7; −1.0)*
Physical disability	2.9 (1.4)	−1.3 (−1.6; −1.0)*	−1.6 (−1.9; −1.2)*
Anxiety	2.7 (1.3)	−1.1 (−1.4; −0.8)*	−1.1 (−1.4; −0.8)*
General well-being	2.2 (1.1)	−0.7 (−1.0; −0.5)*	−0.8 (−1.1; −0.6)*
EQ-5D-5L^a^			
EQ-5D-5L index	0.80 (0.15)	0.06 (0.04; 0.09)*	0.07 (0.04; 0.10)*
EQ-VAS	57.7 (21.0)	11.8 (6.6; 16.9)*	16.1 (10.2; 21.9)*
EQ-5D-5L total score	9.6 (3.3)	−1.6 (−2.1; −1.0)*	−1.6 (−2.3; −0.9)*
EQ-5D-5L sub-scores, mean (SD)			
Mobility	1.5 (0.9)	−0.2 (−0.3; −0.0)*	−0.1 (−0.3; 0.1)
Self-care	1.1 (0.3)	−0.0 (−0.1; 0.0)	−0.0 (−0.1; 0.0)
Usual activities	2.3 (1.1)	−0.4 (−0.7; −0.2)*	−0.5 (−0.8; −0.2)*
Pain/discomfort	2.4 (1.1)	−0.5 (−0.8; −0.3)*	−0.6 (−0.8; −0.3)*
Anxiety/depression	2.3 (1.1)	−0.3 (−0.6; −0.1)*	−0.4 (−0.6; −0.1)*

CR reflects percentage of the whole group with data available for the individual parameter.

a CR >90% for patients with follow-up visits.

* Significant change from baseline using paired *t*-test for mean differences, *p* < 0.05.

CI, confidence interval; CR, coverage rate; EQ-5D-5L, European Quality of Life 5 dimensions 5 levels; EQ-VAS, European Quality of Life – Visual Analogue Scale; SD, standard deviation; SHS, Short Health Scale.

At baseline, EQ-5D-5L demonstrated an impairment mainly in the aspects of pain/discomfort and the ability to participate in common daily activities (Table 3). Improvements in these dimensions were observed both at week 8 and week 16. The overall EQ-5D-5L index also improved significantly from baseline to week 8 and week 16 as did the EQ-VAS, reflecting patients’ overall perception of health (Table 3).

**Figure 5.**
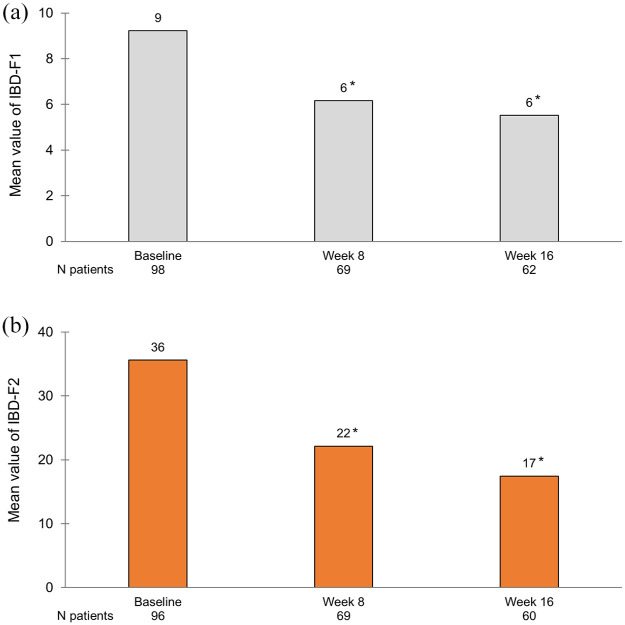
Fatigue as measured by the IBD-F scale, IBD-F1 (a) assessing occurrence and severity, and IBD-F2 (b) assessing impact on daily life, at baseline and follow-up in patients with ongoing tofacitinib treatment. *Significant changes from baseline using paired *t*-test for mean differences (*p* < 0.05). IBD-F, Inflammatory Bowel Disease Fatigue.

Examining the levels of fatigue, significant improvements in the mean IBD-F1 and 2 were seen at weeks 8 and 16 as compared with baseline (Figure 5).

## Discussion

The results from this prospective real-world study corroborate the clinical efficacy of tofacitinib seen in RCTs on patients with UC. Importantly, adding to these observations we present comprehensive data on HRQoL, fatigue, bowel urgency and arthralgia, demonstrating significant effects of tofacitinib on these outcomes.

The clinical efficacy of tofacitinib in UC has been demonstrated in RCTs.^4^ In the OCTAVE study 1 and 2, remission rates of 18.5% and 16.6%, respectively, at week 8 were seen, both of which were significantly higher than the placebo rates (8.2% and 3.6%, respectively). These pivotal studies have then been accompanied by real-world data to ensure the effectiveness and safety of the drug when used in clinical practice in patient populations that may differ from those of the registration trials.^26^ However, a direct comparison between these studies is difficult since factors such as inclusion criteria, outcome definitions and handling of treatment failures and drop-outs differ. Our patient cohort had similar characteristics in terms of age, disease extension and duration of disease as the patient cohorts of the RCTs and previously presented real-world studies.^5,26–33^ In contrast to the OCTAVE trials, where approximately 55% were previously exposed to biologic treatment, 98% of the subjects in our study had been treated with biologic therapies before starting JAKi, reflecting our national guidelines and clinical practice in most healthcare settings. Nevertheless, despite being a more difficult-to-treat group of patients, we found that 33% and 39% had achieved corticosteroid-free remission at weeks 8 and 16, respectively, which is almost twice the numbers in the OCTAVE studies (18.5% and 16.6%). These results are well in line with earlier real-world studies that presented clinical remission rates between 29% and 47%.^5,6,26–29^

Endoscopic data, which was available for approximately half of our patients at week 16, showed that 24% of these had reached endoscopic remission, that is, MES of 0. These figures compare well to a real-world endoscopic study on 148 patients (35% were bio naïve) of which 21% achieved a UC endoscopic index of severity score of 0.^30^ In the absence of complete endoscopic data, we analysed the proportion of patients that displayed strict endoscopic remission (MES 0) or an FC value of <100 mg/kg (used if endoscopic data were missing).^34^ Applying this outcome measure, we found that 38% of patients were in remission at week 16.

Fatigue is a common symptom in IBD with 44%–86% of patients with active disease and 22%–41% of patients in remission suffering from this debilitating disease manifestation.^22,35,36^ Factors such as inflammation, nutritional deficiencies, anaemia, metabolic alterations, microbiota changes, psychological comorbidity, sleep disturbances, muscle dysfunction and physical inactivity are thought to be involved to various degrees in the pathogenesis of IBD-related fatigue. Active IBD and inflammatory activity have been linked to fatigue in numerous studies. Circulating cytokines (including IL-1, IL-6, IL-12, TNFα and IFNγ) are thought to contribute to fatigue through both central effects on the nervous system and peripheral effects on muscles and sensory neurons.^10,12,37^ Nevertheless, fatigue is unmistakably frequent in patients with quiescent disease. The role of subclinical inflammation and cytokines in quiescent disease fatigue is however unclear and data are inconsistent.^38^ The effect of IBD treatment on fatigue is seldom recorded in RCTs and data are thus scarce. Available data however suggest that JAKi therapy may have considerable effects on fatigue in patients with IBD.^39^ Fatigued patients with IBD display aberrations in a wide variety of immune parameters compared to non-fatigued patients.^10^ In the field of inflammatory rheumatic diseases, it has been suggested that JAKi modulate the mechanisms of fatigue in several ways, including (i) decreasing inflammation mediated by cytokines; (ii) inhibiting direct cytokine effects on astrocytes and microglia and (iii) decreasing oxidative stress through the inhibition of indoleamine dioxygenase. It has therefore been proposed that JAK inhibition is a rational approach for treating fatigue, which is strongly supported by our real-world data.

Patients with UC have a well-known deterioration in HRQoL^40^ and correlations with disease activity, gender and comorbidity have been observed.^41^ Previous prospective real-world studies have addressed this,^5,29^ but our study presents more comprehensive data on HRQoL and fatigue. We observed a marked improvement in measures of quality of life using SHS and EQ-VAS, as well as a reduction in the occurrence and severity of fatigue by one-third as measured by the IBD-F scale. An association between clinical disease activity and fatigue has previously been established^42^ but also IBD patients in remission have been shown to have higher levels of fatigue than healthy controls.^35^ There are studies indicating that endoscopic activity and fatigue are not always linked, further emphasizing the importance of studies beyond intestinal disease activity when evaluating drug efficacy.^43^

Bowel urgency has been highlighted as a sensitive and feasible clinical measure for determining the degree of inflammatory activity but also as a factor that has a major impact on quality of life.^44^ For patients with UC, the insecurity of not being able to trust their bowel movements and potential faecal incontinence are major stress factors. As a result, patients constantly keep track of where the nearest toilet is located, and mobility and travel may therefore be restricted. In our study, 85% of patients had bowel urgency at inclusion. Already after 2 weeks of tofacitinib treatment, we observed a marked decrease in this number, and at week 16 a reduction in the bowel urgency score of more than half was observed. Urgency and especially incontinence rank as highly distressing symptoms, and it is thus very plausible that the decreased levels of bowel urgency are directly linked to the observed improvements in HRQoL.

The broad immunologic effects of tofacitinib may be beneficial for patients with systemic signs of their UC such as EIMs. The intensity of systemic disease manifestations often covary with the level of intestinal inflammation but can also be independent. Musculoskeletal EIMs represent a major concern in patients with IBD and depending on definition, the reported frequency differs vastly between 6% and 46%.^45^ Patients with IBD-associated arthropathy have an increased risk of reduced work productivity compared to other IBD patients, causing impairments in HRQoL and economic disadvantages.^46^ Primary studies on EIMs in IBD are limited and drugs that have shown efficacy in the primary diseases in the affected organ system are often used instead. Tofacitinib is approved for rheumatoid arthritis, psoriatic arthritis and spondyloarthritis. Whether the inflammatory mechanisms are shared in non-IBD arthritis and IBD-associated arthropathy is unclear. Circulating immune complexes due to the phenomenon of a leaky gut is one model to explain the association with inflammatory activity in the gut. It has also been hypothesized that T-lymphocytes activated in the gut may recirculate to joints and adhere to synovial tissue.^47^ Moreover, musculoskeletal EIMs have been shown to be associated with genetic polymorphisms in the NOD2 and IL-23 genes, and with specific HLA genotypes.^45^ Our finding that 62% of the patients that had arthralgia at inclusion did not report arthralgia following the start of tofacitinib treatment indicates a meaningful impact on this symptom. Arthritis is typically related to disease activity. As tofacitinib has a broad immunological effect through the inhibition of the JAK/STAT signalling pathway, a beneficial effect on IBD-associated arthropathy is theoretically plausible.

Most patients (62%) in our study did not de-escalate to 5 mg twice daily or had re-escalated by week 16 which makes the results difficult to compare to previous studies, although recent observational studies demonstrate a similar trend with 40% of patients on 10 mg twice daily at follow-up.^6^ Considering that high endoscopic activity and high CRP levels at inclusion correlate with lower response rates^27,28,30^ and nearly half of patients do not regain response after re-escalation^27,28^ it may be a rational approach to keep the higher dose in patients with more severe initial inflammation. The increasing use of higher JAKi doses may reflect a growing clinical experience of the dose-dependent effects of JAKis. Furthermore, since the population receiving JAKi is often highly treatment experienced, tofacitinib may in some cases be the last available treatment before colectomy.

The landscape of treatment options for UC is rapidly changing, and so are the ambitions regarding treatment goals for our UC patients. Having previously set the goal at improvement of symptoms, we now also aim for endoscopic healing (and potentially histologic healing) and restoration of quality of life. In addition, we aim to reach these goals with the patient weaned from corticosteroids and kept corticosteroid-free. The road to these goals however is still uncertain; the high proportion of patients in our cohort exposed to multiple advanced therapies prior to inclusion and the high percentage of corticosteroid use at inclusion strongly indicates frequent failure to reach therapeutic goals. Furthermore, symptoms directly related to colonic inflammation are often reported to the treating physician, while EIMs and more diffusely associated symptoms such as fatigue may be brought up less often by the patient and likely in general receive less attention from the treating physician. The association between IBD and fatigue is well described^42^ but being tired is a common condition and the border to pathological fatigue is sometimes difficult to identify. Fatigue may be stigmatizing, and it has been reported that patients are unwilling to describe their fatigue to their physician in the absence of effective solutions.

There are several weaknesses in our study, many related to the observational real-world setting with follow-up not strictly regulated by the protocol. This results in the coverage of not all data being complete at given follow-up timepoints. Although coverage of endoscopic data was inconsistent with large variations between centres and the possibility that refractory patients may have been exposed to endoscopy more frequently, we have chosen to present the available data. The window for follow-up was wide, (±4 at week 16) also due to the observational character of the study. Although inclusion criteria were well defined, patients considered for inclusion may not have been all patients from including centres, decision was based on a clinical judgement which is a weakness. Another weakness is the use of local laboratories resulting in a variation of laboratory testing methods. Hb and CRP are well harmonized across Sweden, but calprotectin has a substantial inconsistency depending on the analyzing method. We have tried to minimize the impact by mainly presenting relative changes and cuts of values for remission. We have also ensured that all the patients’ individual patients were followed by the same method over time. Data on HRQoL and fatigue covers only patients still on tofacitinib and may not represent the study population at large. The strength of this study is the prospectively followed cohort which enables the presentation of comprehensive data concerning several important aspects of UC and their impact on patients’ lives. We have used objective outcome measures of disease activity including FC, CRP and endoscopy which together with prospective patient-reported outcome measures of disease activity, EIMs, quality of life and measures of fatigue gives a comprehensive and detailed understanding of the therapeutic effects of tofacitinib.

In conclusion, we observed that induction therapy with tofacitinib was associated with improvements in patient-reported outcome measures of symptoms, arthralgia, HRQoL and fatigue as well as endoscopic activity. These real-world data indicate that tofacitinib is a fast-acting and effective drug for treating UC and UC-associated morbidity.

## Supplemental Material

sj-doc-1-tag-10.1177_17562848251343427 – Supplemental material for Prospective observational study of tofacitinib in ulcerative colitis – analysis of clinical data, fatigue and health-related quality of life during the induction phase Supplemental material, sj-doc-1-tag-10.1177_17562848251343427 for Prospective observational study of tofacitinib in ulcerative colitis – analysis of clinical data, fatigue and health-related quality of life during the induction phase by Lisa Nyberg, Jonas Halfvarson, Jonas Söderling, Ola Olén, Hans Strid, Charlotte R. H. Hedin, Sara B. Jónsdóttir, Henrik Hjortswang, Susanna Jäghult, Joseph C. Cappelleri, Dan Henrohn, Maria Seddighzadeh, Jan Marsal and Olof Grip in Therapeutic Advances in Gastroenterology

## Appendix

### Abbreviations

CI confidence interval

CRP C-reactive protein

eCRF electronic case report form

EIM extraintestinal manifestation

EQ-5D-5L European Quality of life 5 dimensions 5 levels

EQ-VAS European Quality of Life – Visual analogue scale

FC faecal calprotectin

HRQoL health-related quality of life

IBD inflammatory bowel disease

IBD-F inflammatory bowel disease fatigue

IQR inter quartile range

JAK Janus kinase

JAKi Janus kinase inhibitor

MES Mayo Endoscopic Score

NA not applicable

NR not reported

ODEN observational study of tofacitinib in ulcerative colitis in Sweden

PGA physician global assessment

p-Mayo partial Mayo

PRO-2 two item patient-reported outcome

RCT randomized controlled trial

SCCAI simple clinical colitis activity index

SHS Short Health Scale

SmPC summary of product characteristics

SWIBREG Swedish Inflammatory Bowel Disease Registry

TNF tumour necrosis factor

UC ulcerative colitis

## Declarations

**Ethics approval and consent to participate:** The study was approved by the Regional Ethics Review Board in Uppsala, Etikprövningsmyndigheten, Sweden, registry number 2020-01346. The clinical decision to initiate tofacitinib therapy was clearly separated from the option to be included in the study. Proposed follow-up timepoints were designed to harmonize with standard care in order not to expose the patients to unnecessary tests or investigations. Proposed follow-up timepoints were designed to harmonize with standard care in order not to expose the patients to unnecessary tests or investigations. Consent to participate: All patients provided written informed consent.

**Consent for publication:** Not applicable.

**Availability of data and materials:** Data is available on request.

## References

[1] Raine T Bonovas S Burisch J , et al. ECCO guidelines on therapeutics in ulcerative colitis: medical treatment. J Crohns Colitis 2022; 16(1): 2–17. 34635919 10.1093/ecco-jcc/jjab178

[2] Turner D Ricciuto A Lewis A , et al. STRIDE-II: an update on the selecting therapeutic targets in inflammatory bowel disease (STRIDE) initiative of the international organization for the study of IBD (IOIBD): determining therapeutic goals for treat-to-target strategies in IBD. Gastroenterology 2021; 160(5): 1570–1583. 33359090 10.1053/j.gastro.2020.12.031

[3] Feuerstein JD Isaacs KL Schneider Y , et al. AGA clinical practice guidelines on the management of moderate to severe ulcerative colitis. Gastroenterology 2020; 158(5): 1450–1461. 31945371 10.1053/j.gastro.2020.01.006 PMC7175923

[4] Sandborn WJ Su C Sands BE , et al. Tofacitinib as induction and maintenance therapy for ulcerative colitis. N Engl J Med 2017; 376(18): 1723–1736. 28467869 10.1056/NEJMoa1606910

[5] Long MD Afzali A Fischer M , et al. Tofacitinib response in ulcerative colitis (TOUR): early response after initiation of tofacitinib therapy in a real-world setting. Inflamm Bowel Dis 2023; 29(4): 570–578. 35700276 10.1093/ibd/izac121 PMC10069660

[6] Chaparro M Acosta D Rodríguez C , et al. Real-world evidence of tofacinitib in ulcerative colitis: short-term and long-term effectiveness and safety. Am J Gastroenterol 2023; 118(7): 1237–1247. 36716287 10.14309/ajg.0000000000002145

[7] Armuzzi A Hart A Cappelleri JC , et al. Characteristics, clinical outcomes and patient-reported outcomes of patients with ulcerative colitis receiving tofacitinib: a real-world survey in the United States and five European countries. BMC Gastroenterol 2023; 23(1): 17. 36658481 10.1186/s12876-023-02640-7 PMC9849840

[8] Salas A Hernandez-Rocha C Duijvestein M , et al. JAK-STAT pathway targeting for the treatment of inflammatory bowel disease. Nat Rev Gastroenterol Hepatol 2020; 17(6): 323–337. 32203403 10.1038/s41575-020-0273-0

[9] Rubin DT Reinisch W Greuter T , et al. Extraintestinal manifestations at baseline, and the effect of tofacitinib, in patients with moderate to severe ulcerative colitis. Therap Adv Gastroenterol 2021; 14: 17562848211005708. 10.1177/17562848211005708 PMC8132089 34035832

[10] McGing JJ Radford SJ Francis ST , et al. Review article: The aetiology of fatigue in inflammatory bowel disease and potential therapeutic management strategies. Aliment Pharmacol Ther 2021; 54(4): 368–387. 34228817 10.1111/apt.16465

[11] Christensen KR Ainsworth MA Steenholdt C , et al. Fatigue is a systemic extraintestinal disease manifestation largely independent of disease activity, chronicity, and nutritional deficiencies in inflammatory bowel disease on biologics. Scand J Gastroenterol 2022; 57(9): 1051–1057. 35412932 10.1080/00365521.2022.2060049

[12] Borren NZ van der Woude CJ Ananthakrishnan AN . Fatigue in IBD: epidemiology, pathophysiology and management. Nat Rev Gastroenterol Hepatol 2019; 16(4): 247–259. 30531816 10.1038/s41575-018-0091-9

[13] von Elm E Altman DG Egger M , et al. The Strengthening the Reporting of Observational Studies in Epidemiology (STROBE) statement: guidelines for reporting observational studies. Lancet 2007; 370(9596): 1453–1457. 18064739 10.1016/S0140-6736(07)61602-X

[14] Naegeli AN Hunter T Dong Y , et al. Full, partial, and modified permutations of the Mayo score: characterizing clinical and patient-reported outcomes in ulcerative colitis patients. Crohns Colitis 360 2021; 3(1): otab007. 10.1093/crocol/otab007 PMC9802037 36777063

[15] Jairath V Khanna R Zou GY , et al. Development of interim patient-reported outcome measures for the assessment of ulcerative colitis disease activity in clinical trials. Aliment Pharmacol Ther 2015; 42(10): 1200–1210. 26388424 10.1111/apt.13408

[16] Walmsley RS Ayres RC Pounder RE , et al. A simple clinical colitis activity index. Gut 1998; 43(1): 29–32. 9771402 10.1136/gut.43.1.29 PMC1727189

[17] Hjortswang H Järnerot G Curman B , et al. The Short Health Scale: a valid measure of subjective health in ulcerative colitis. Scand J Gastroenterol 2006; 41(10): 1196–1203. 16990205 10.1080/00365520600610618

[18] Latteur J Ernstsson O Nilsson E , et al. Construct validity of EQ-5D-5L among patients with inflammatory bowel disease – a study based on real-world data from the Swedish Inflammatory Bowel Disease Registry. J Patient Rep Outcomes 2024; 8(1): 39. 38536626 10.1186/s41687-024-00709-9 PMC10973303

[19] Sun S Chuang LH Sahlén KG , et al. Estimating a social value set for EQ-5D-5L in Sweden. Health Qual Life Outcomes 2022; 20(1): 167. 36564844 10.1186/s12955-022-02083-w PMC9780618

[20] Stark RG Reitmeir P Leidl R , et al. Validity, reliability, and responsiveness of the EQ-5D in inflammatory bowel disease in Germany. Inflamm Bowel Dis 2010; 16(1): 42–51. 19475674 10.1002/ibd.20989

[21] Coteur G Feagan B Keininger DL , et al. Evaluation of the meaningfulness of health-related quality of life improvements as assessed by the SF-36 and the EQ-5D VAS in patients with active Crohn’s disease. Aliment Pharmacol Ther 2009; 29(9): 1032–1041. 19222413 10.1111/j.1365-2036.2009.03966.x

[22] Czuber-Dochan W Norton C Bassett P , et al. Development and psychometric testing of inflammatory bowel disease fatigue (IBD-F) patient self-assessment scale. J Crohns Colitis 2014; 8(11): 1398–1406. 24856864 10.1016/j.crohns.2014.04.013

[23] Eriksson C Rundquist S Lykiardopoulos V , et al. Real-world effectiveness of vedolizumab in inflammatory bowel disease: week 52 results from the Swedish prospective multicentre SVEAH study. Therap Adv Gastroenterol 2021; 14: 17562848211023386. 10.1177/17562848211023386 PMC8255566 34276808

[24] Forss A Clements M Myrelid P , et al. Ustekinumab is associated with real-world long-term effectiveness and improved health-related quality of life in Crohn’s disease. Dig Dis Sci 2023; 68(1): 65–76. 35459973 10.1007/s10620-022-07501-z PMC9883312

[25] Rosner B . Fundamentals of biostatistics. 8th ed. Boston, MA: Cengage Learning, 2015.

[26] Taxonera C Olivares D Alba C . Real-world effectiveness and safety of tofacitinib in patients with ulcerative colitis: systematic review with meta-analysis. Inflamm Bowel Dis 2022; 28(1): 32–40. 33586766 10.1093/ibd/izab011

[27] Ma C Panaccione R Xiao Y , et al. REMIT-UC: real-world effectiveness and safety of tofacitinib for moderate-to-severely active ulcerative colitis: a Canadian IBD research consortium multicenter national cohort study. Am J Gastroenterol 2023; 118(5): 861–871. 36580497 10.14309/ajg.0000000000002129 PMC10144270

[28] Honap S Chee D Chapman TP , et al. Real-world effectiveness of tofacitinib for moderate to severe ulcerative colitis: a multicentre UK experience. J Crohns Colitis 2020; 14(10): 1385–1393. 32280965 10.1093/ecco-jcc/jjaa075

[29] Biemans VBC Sleutjes JAM de Vries AC , et al. Tofacitinib for ulcerative colitis: results of the prospective Dutch Initiative on Crohn and Colitis (ICC) registry. Aliment Pharmacol Ther 2020; 51(9): 880–888. 32237087 10.1111/apt.15689 PMC7187329

[30] Shin SH Oh K Hong SN , et al. Real-life effectiveness and safety of tofacitinib treatment in patients with ulcerative colitis: a KASID multicenter cohort study. Therap Adv Gastroenterol 2023; 16: 17562848231154103. 10.1177/17562848231154103 PMC10026122 36950251

[31] Lair-Mehiri L Stefanescu C Vaysse T , et al. Real-world evidence of tofacitinib effectiveness and safety in patients with refractory ulcerative colitis. Dig Liver Dis 2020; 52(3): 268–273. 31732444 10.1016/j.dld.2019.10.003

[32] Molander P Kosunen M Eronen H , et al. Tofacitinib real-world experience in ulcerative colitis in Finland (FinTofUC): a retrospective non-interventional multicenter patient chart data study. Scand J Gastroenterol 2024; 59(4): 425–432. 38156792 10.1080/00365521.2023.2298361

[33] Lucaciu LA Constantine-Cooke N Plevris N , et al. Real-world experience with tofacitinib in ulcerative colitis: a systematic review and meta-analysis. Therap Adv Gastroenterol 2021; 14: 17562848211064004. 10.1177/17562848211064004 PMC8721385 34987608

[34] Buisson A Mak WY Andersen MJ , et al. Faecal calprotectin is a very reliable tool to predict and monitor the risk of relapse after therapeutic de-escalation in patients with inflammatory bowel diseases. J Crohns Colitis 2019; 13(8): 1012–1024. 30726887 10.1093/ecco-jcc/jjz023 PMC6939876

[35] van Langenberg DR Gibson PR . Systematic review: fatigue in inflammatory bowel disease. Aliment Pharmacol Ther 2010; 32(2): 131–143. 20456309 10.1111/j.1365-2036.2010.04347.x

[36] Bager P Vestergaard C Juul T , et al. Population-based normative data for the inflammatory bowel disease fatigue scale – IBD-F. Scand J Gastroenterol 2018; 53(10–11): 1274–1279. 30351212 10.1080/00365521.2018.1521868

[37] Vogelaar L de Haar C Aerts BR , et al. Fatigue in patients with inflammatory bowel disease is associated with distinct differences in immune parameters. Clin Exp Gastroenterol 2017; 10: 83–90. 28496351 10.2147/CEG.S123942 PMC5422327

[38] Qazi T . Fatigue in inflammatory bowel disease: a problematic ailment. Curr Opin Gastroenterol 2020; 36(4): 284–294. 32398564 10.1097/MOG.0000000000000644

[39] Ghosh S Feagan BG Parra RS , et al. Impact of upadacitinib induction and maintenance therapy on health-related quality of life, fatigue, and work productivity in patients with moderately-to-severely active Crohn’s disease. J Crohns Colitis 2024; 18(11): 1804–1818. 38835235 10.1093/ecco-jcc/jjae083 PMC11532615

[40] Knowles SR Graff LA Wilding H , et al. Quality of life in inflammatory bowel disease: a systematic review and meta-analyses – Part I. Inflamm Bowel Dis 2018; 24(4): 742–751. 29562277 10.1093/ibd/izx100

[41] Hjortswang H Järnerot G Curman B , et al. The influence of demographic and disease-related factors on health-related quality of life in patients with ulcerative colitis. Eur J Gastroenterol Hepatol 2003; 15(9): 1011–1020. 12923375 10.1097/00042737-200309000-00012

[42] Amiot A Chaibi S Bouhnik Y , et al. Prevalence and determinants of fatigue in patients with IBD: a cross-sectional survey from the GETAID. J Crohns Colitis 2023; 17(9): 1418–1425. 36988620 10.1093/ecco-jcc/jjad060

[43] Grimstad T Skjellerudsveen BM Kvaløy JT , et al. The influence of disease activity on fatigue in patients with ulcerative colitis – a longitudinal study. Scand J Gastroenterol 2022; 57(3): 290–297. 34846950 10.1080/00365521.2021.2007281

[44] D’Haens G Dubinsky M Kobayashi T , et al. Mirikizumab as induction and maintenance therapy for ulcerative colitis. N Engl J Med 2023; 388(26): 2444–2455. 37379135 10.1056/NEJMoa2207940

[45] Rogler G Singh A Kavanaugh A , et al. Extraintestinal manifestations of inflammatory bowel disease: current concepts, treatment, and implications for disease management. Gastroenterology 2021; 161(4): 1118–1132. 34358489 10.1053/j.gastro.2021.07.042 PMC8564770

[46] van der Have M Brakenhoff LK van Erp SJ , et al. Back/joint pain, illness perceptions and coping are important predictors of quality of life and work productivity in patients with inflammatory bowel disease: a 12-month longitudinal study. J Crohns Colitis 2015; 9(3): 276–283. 25547976 10.1093/ecco-jcc/jju025

[47] Hedin CRH Vavricka SR Stagg AJ , et al. The pathogenesis of extraintestinal manifestations: implications for IBD research, diagnosis, and therapy. J Crohns Colitis 2019; 13(5): 541–554. 30445584 10.1093/ecco-jcc/jjy191

