# Supplementary material for: Prospective observational study of tofacitinib in ulcerative colitis – analysis of clinical data, fatigue and health-related quality of life during the induction phase
Source: Ther Adv Gastroenterol. 2025 Jun 16;18:17562848251343427. doi: 10.1177/17562848251343427 (PMC12174757; doi:10.1177/17562848251343427)
Supplement: sj-doc-1-tag-10.1177_17562848251343427 – Supplemental material for Prospective observational study of tofacitinib in ulcerative colitis – analysis of clinical data, fatigue and health-related quality of life during the induction phase [file sj-doc-1-tag-10.1177_17562848251343427.doc]

STROBE Statement—Checklist of items that should be included in reports of ***cohort studies***

|  | Item No | Recommendation |
| --- | --- | --- |
| **Title and abstract** | 1 | (*a*) Indicate the study’s design with a commonly used term in the title or the abstract  Title and abstract separate |
|  |  | (*b*) Provide in the abstract an informative and balanced summary of what was done and what was found Abstract separate |
| Introduction | | |
| Background/rationale | 2 | Explain the scientific background and rationale for the investigation being reported  Page 1 |
| Objectives | 3 | State specific objectives, including any prespecified hypotheses  Page 1 |
| Methods | | |
| Study design | 4 | Present key elements of study design early in the paper  Page 1-2 |
| Setting | 5 | Describe the setting, locations, and relevant dates, including periods of recruitment, exposure, follow-up, and data collection  Page 1-2, figure 5 timeline |
| Participants | 6 | (*a*) Give the eligibility criteria, and the sources and methods of selection of participants. Describe methods of follow-up  Page2-3 |
|  |  | (*b*) For matched studies, give matching criteria and number of exposed and unexposed  *NA* |
| Variables | 7 | Clearly define all outcomes, exposures, predictors, potential confounders, and effect modifiers. Give diagnostic criteria, if applicable  Page 2-3 |
| Data sources/ measurement | 8* | For each variable of interest, give sources of data and details of methods of assessment (measurement). Describe comparability of assessment methods if there is more than one group  Page 2-3 |
| Bias | 9 | Describe any efforts to address potential sources of bias  Page 3, statistics |
| Study size | 10 | Explain how the study size was arrived at  Page 2 patient population |
| Quantitative variables | 11 | Explain how quantitative variables were handled in the analyses. If applicable, describe which groupings were chosen and why  Page 4 |
| Statistical methods | 12 | (*a*) Describe all statistical methods, including those used to control for confounding Page 4 |
|  |  | (*b*) Describe any methods used to examine subgroups and interactions  NA |
|  |  | (*c*) Explain how missing data were addressed  Page 4 |
|  |  | (*d*) If applicable, explain how loss to follow-up was addressed  Page 4 |
|  |  | (*e*) Describe any sensitivity analyses  NA |
| Results | | |
| Participants | 13* | (a) Report numbers of individuals at each stage of study—eg numbers potentially eligible, examined for eligibility, confirmed eligible, included in the study, completing follow-up, and analysed  Page 4-5, Figures and tables |
|  |  | (b) Give reasons for non-participation at each stage Page 4 |
|  |  | (c) Consider use of a flow diagram Figure |
| Descriptive data | 14* | (a) Give characteristics of study participants (eg demographic, clinical, social) and information on exposures and potential confounders Table 1 |
|  |  | (b) Indicate number of participants with missing data for each variable of interest Page 4-5, figures and tables |
|  |  | (c) Summarise follow-up time (eg, average and total amount) Page 2 Statistics |
| Outcome data | 15* | Report numbers of outcome events or summary measures over time NA |
| Main results | 16 | (*a*) Give unadjusted estimates and, if applicable, confounder-adjusted estimates and their precision (eg, 95% confidence interval). Make clear which confounders were adjusted for and why they were included  Page 4-6, tables and figures |
|  |  | (*b*) Report category boundaries when continuous variables were categorized  Page 2-3 |
|  |  | (*c*) If relevant, consider translating estimates of relative risk into absolute risk for a meaningful time period  NA |
| Other analyses | 17 | Report other analyses done—eg analyses of subgroups and interactions, and sensitivity analyses  NA |
| Discussion | | |
| Key results | 18 | Summarise key results with reference to study objectives  Page 4-6, Table 1-3 and figures 1-4 |
| Limitations | 19 | Discuss limitations of the study, taking into account sources of potential bias or imprecision. Discuss both direction and magnitude of any potential bias  Page 8 |
| Interpretation | 20 | Give a cautious overall interpretation of results considering objectives, limitations, multiplicity of analyses, results from similar studies, and other relevant evidence  Page 6-9 |
| Generalisability | 21 | Discuss the generalisability (external validity) of the study results  Page 9 |
| Other information | | |
| Funding | 22 | Give the source of funding and the role of the funders for the present study and, if applicable, for the original study on which the present article is based  Page 11 |

*Give information separately for exposed and unexposed groups.

**Note:** An Explanation and Elaboration article discusses each checklist item and gives methodological background and published examples of transparent reporting. The STROBE checklist is best used in conjunction with this article (freely available on the Web sites of PLoS Medicine at http://www.plosmedicine.org/, Annals of Internal Medicine at http://www.annals.org/, and Epidemiology at http://www.epidem.com/). Information on the STROBE Initiative is available at http://www.strobe-statement.org.
